# Perioperative analgesic management using conventional versus regional nerve block in craniotomy surgeries in lower middle-income countries

**DOI:** 10.12669/pjms.42.9.19519

**Published:** 2026-09

**Authors:** Dileep Kumar, Aqsa Rasheed, Mohsin Nazir

**Affiliations:** 1 Dileep Kumar, Associate Professor, Department of Anesthesia, The Aga Khan University Hospital, Karachi 74800, Pakistan; 2 Aqsa Rasheed, Resident Trainee, Department of Anesthesia, The Aga Khan University Hospital, Karachi 74800, Pakistan; 3 Mohsin Nazir, Associate Professor, Department of Anesthesia, The Aga Khan University Hospital, Karachi 74800, Pakistan

**Keywords:** Anesthesia, Craniotomy, Morphine, Neurosurgery, Pain, Scalp Nerve Block

## Abstract

**Objectives:**

This study’s primary objective was to assess the efficacy of systemic opioids with pre-incision bilateral scalp nerve block for supratentorial craniotomy pain via intraoperative hemodynamic variables at different intervals. The secondary objective was to assess the postoperative pain, rescue analgesia requirement, sedation, nausea and vomiting in first 24 hours of surgery.

**Methodology:**

This double-blinded randomized controlled trial, conducted at Aga Khan University Hospital, Karachi. The 62 adult patients (ASA I–III), recruited for elective supratentorial craniotomy via randomization into two groups (n=31/group). Group-M received intravenous morphine (0.1 mg/kg) at induction. Group-S received bilateral SNB with lidocaine, ropivacaine, adrenaline, and dexamethasone. The heart rate, systolic, diastolic, and mean arterial pressures were recorded at predefined intraoperative intervals. Postoperatively, both participants received PCIA morphine, and pain was assessed by visual analogue scale (VAS), along with sedation, nausea and vomiting incidences, which were recorded in first 24 hours at various intervals.

**Results:**

The demographic and surgical characteristics were insignificant in both groups (*p*<0.05). The hemodynamic variables such as heart rate was only increased on intense stimulation in both groups, relatively was higher in group-S compared to group-M. However, statistically it was insignificant. The systolic, diastolic, and mean arterial pressures were significantly stable in group-S, compared to Group-M, at various intraoperative intervals (*p*<0.05). The sedation, nausea and vomiting incidence were equivocal in both groups. The VAS pain score was significantly lower in group-S, compared to group-M, postoperatively from 2 to 24 hours.

**Conclusion:**

Pre-incisional scalp nerve block provides better attenuation of intraoperative noxious stimuli via hemodynamic control, and it shows superior postoperative analgesia, without risking the sedation, nausea and vomiting in supratentorial craniotomy surgeries.

## INTRODUCTION

Optimal pain management attenuates intraoperative and postoperative surgical stress response and offers hemodynamic stability in craniotomy surgeries.^1^ More than 55% of patients have experienced moderate to severe pain within the first 24 hours following craniotomy surgeries.^2^ The literature is evident with almost 25% incidence of inadequate perioperative craniotomy pain management^3^ and it is associated with serious short to long-term complications, such as intraoperative cerebral hyperemia, brain swelling,^4^ postoperative intracranial hemorrhage,^5^ agitation, and the development of chronic pain syndromes. Despite extensive research, no consensus exists regarding the most effective perioperative analgesic strategy for elective brain tumor surgeries.^3^ Multimodal analgesic regimen such as paracetamol, NSAIDs, dexmedetomidine and incision-site infiltration are also in practice.^6^ However, opioid remains the mainstay for managing moderate to severe pain in craniotomy surgical patients.^7^ Nevertheless, systemic opioid administration is also associated with adverse effects such as nausea, vomiting, respiratory depression, and over-sedation, which may interfere with neurological examination and delay the detection of surgical complications.^8^ Furthermore the scalp nerve block (SNB) is a long-established alternative to systemic pharmacologic intervention for craniotomy pain. It has demonstrated potential benefits when performed prior to surgical incision,^9^ like attenuation of hemodynamic responses to noxious stimuli, improved intraoperative stability, smoothen emergence,^10^ reduces the postoperative pain score,^11^ and minimizes the opioid consumption and it’s side effects.^12^ Although the local data is scarce on perioperative craniotomy pain in our region, effective pain management is an existing challenge because of uncertainty in the availability of opioids and expertise. Therefore, we devised our study to test the SNB as a substitute for craniotomy pain.

The primary objective of our study was to compare systemic opioids with pre-incision bilateral scalp nerve block for perioperative supratentorial craniotomy pain management. The secondary objective was to assess the postoperative pain, rescue analgesia requirement, sedation scores, and the incidence of nausea and vomiting in first 24 hours of surgery.

## METHODOLOGY

This double blinded, randomized controlled trial was conducted at The Aga Khan University Hospital, Karachi, from January 2025 to July 2025.

### Ethical approval:

Institutional Ethical Review Committee was taken from The Aga Khan University Hospital, Karachi (ERC reference number - 2025-9126-34025) which was obtained in April 5, 2024. Protocol was registered at clinicaltrials.gov (NCT07425678). The written informed consent was taken from 62 adult patients aged 18-75 years, ASA I-III, scheduled for elective supratentorial craniotomy.

The patients having Glasgow Coma Score less than 15, known allergic to study drugs or on chronic narcotic usage, liver dysfunction and inability to comprehend Visual Analogue Scale (VAS) were excluded. All study patients were educated preoperatively for pain assessment using the visual analogue scale (VAS) scores 0-10, (0, no pain and 10 worst pain), and to operate patient-controlled analgesia (PCA) machine for postoperative pain management. Our study used non-probability consecutive computer-generated randomization sampling technique by dividing the study participants into two groups (n=31/group), Group-M (morphine) and Group-S (scalp nerve block). The patient’s study parameters were collected by an independent data collector assigned in the operating room and postoperatively by a pain nurse. The data collector and patient, both were blinded to the study groups and coding was used for documentation to ensure data blinding.

The study began with applying the standard ASA monitors (ECG, NIBP, SPO_2_), and baseline heart rate and blood pressure were recorded. The Oro-nasal temperature probe was used for temperature monitoring, and arterial line was placed in radial artery after the induction of anesthesia in all patients. The anesthesia induction was standardized in both groups with propofol 2mg/kg, and cis-atracurium 0.15mg/kg. Group-M received intravenous morphine (0.1mg/kg) at induction and Group-S, received the intravenous fentanyl 1.5-2mcg/kg to blunt intubation response prior to SNB. The scalp nerve block was performed by primary investigator experienced in SNB, using a mixture of lidocaine 0.1%, ropivacaine 0.25%, adrenaline 1:200,000, and dexamethasone 4mg. Each nerve infiltrated with 2-3-mls of mixture volume bilaterally at supraorbital, supratrochlear, auriculotemporal, zygomaticotemporal, greater, and lesser occipital nerve. The anesthesia was maintained in both groups with the isoflurane (MAC 0.7-1.2), in a mixture of oxygen & nitrous oxide 50% along with cis-atracurium infusion at rate of 0.03 mg kg^-1^ per hour. All patients received intravenous paracetamol 1gram and intravenous dexamethasone 0.15-mg/kg at induction of anesthesia and local anesthesia (1% lidocaine with 1:200000 adrenaline), was infiltrated at Mayfield frame pin sites and on incision line.

The primary objective of study was the intraoperative pain assessment by recording the noxious stimuli induced hemodynamic variations; heart rate, systolic, diastolic, and mean arterial blood pressures response at different time intervals like on intubation, three minutes after intubation, prior to scalp nerve block, three minutes after the scalp nerve block, before head pinning, one and three minutes after the head pinning, at surgical incision, three minutes after skin incision, during bone and periosteum dissection, Dural opening, Dural closing, skull bone closure and at skin closure. The study device to intervene at fluctuation of mean arterial pressure or heart rate greater than 20% of baseline values to initially managed with the increasing of isoflurane end-tidal alveolar concentration up to a MAC of 1.2. Secondly, when mean arterial pressure and heart rate remained elevated, a rescue analgesic bolus of fentanyl 0.5µg/kg was administered in both study groups. No specific end-tidal CO_2_ target was predefined; ventilation was managed to maintain the end-tidal CO_2_ within the standard range. Both study groups received intravenous ondansetron 4-8 mg prior to end of surgery for emesis prophylaxis and muscle relaxation was reversed with neostigmine 0.04mg kg^-1^and glycopyrrolate 0.006mg kg^-1^.

The secondary objective was to assess the postoperative pain scores using the VAS scale along with rescue analgesic requirement, sedation scores, and the incidence of nausea and vomiting. The patients pain score was assessed at various time intervals, upon arrival in PACU, at 30 minutes, 1, 2, 4, 6, 12, 18, 24 hours. Patients were also monitored for sedation score on four-point scale (1 = awake and communicative, 2 = asleep, responds to normal speech, 3 = asleep, responds to pain, 4 = deeply sedated), and nausea vomiting score at four-point scale (0 = no nausea and vomiting, 1 = mild nausea, 2 = severe nausea with retching, 3 = vomiting), for 24 hours postoperatively.

Postoperatively, study participants received patient control analgesia (PCA), morphine (1mg/ml) in PACU for 24 hours at a setting of 1-mg bolus, 10 minutes lockout, and zero background. At the initiation of the study, postoperative morphine was available through PCA; however, during the study period, PCA morphine availability became intermittent because of supply limitations, and PCA could therefore not be provided to all participants. Consequently, postoperative morphine consumption data was incomplete and could not be reliably compared between the study groups; this limitation is addressed further in the discussion.

The sample size was calculated using the STATA version 14.0 software. A test (two sided), comparing two independent means was used. The estimate was based on the study, the baseline mean±SD of MAP (mmHg) for Group-S was 68.9±4.0 while Group-M was 72.2 ± 3.6. that with a 10% drop rate, 24 patients in each group would be required, using a 5% α (level of significance) and 20% β (power of test) with a confidence interval of 95%. It was also estimated that 28 patients in each group would be required to achieve 80% power at the 5% significance level to demonstrate a difference of 50% on the VAS pain score, assuming that VAS mean±SD for the first 24 hours after surgery would be 4.1±3.1 for the control group and 1.9±2.6 for the intervention group. To reduce the impact of data loss, we enrolled 31 patients in each group, assuming a 10% dropout rate. The highest sample size (31 patients per group) was decided to recruit the participants in the study. Sample size was calculated for both the primary hemodynamic outcome (MAP) and the postoperative pain outcome (VAS): the MAP-based calculation yielded a required sample size of 24 patients per group, whereas the VAS-based calculation yielded 28 patients per group. The higher of the two requirements, based on postoperative VAS, was therefore used as the determinant of the final sample size, and 31 patients per group were enrolled to provide an additional margin above this calculated requirement.

### Statistical Analysis:

The R software (version 4.2.1, R Foundation for Statistical Computing, Vienna, Austria) was used to analyze the data. A comparative analysis was conducted between Group-M (Systemic Morphine) and Group-S (Intervention) to determine any statistically significant differences. The normality assumption for numerical variables, such as age, weight, height, duration of surgery and anesthesia, intraoperative hemodynamics and postoperative numerical variables, and VAS scoring, etc, was tested using the Shapiro-Wilk test. For normally (non-normally) distributed variables, the mean±standard deviation (median± interquartile range) was calculated and was analyzed between groups using an unpaired independent t-test (Mann-Whitney). Categorical data, such as sex, diagnosis, type of craniotomy, and craniotomy incision, was stated using frequency and percentage and compared using either the Chi-square or Fisher’s exact test. A significance level of P ≤0.05 was used.

## RESULTS

A total of **62 patients** recruited in both groups, Group-M & Group-S (n=31/group), for supratentorial craniotomy surgeries. There was **no dropout** and all patients were included in the final analysis (Fig.1). Patient demographics, age, sex, weight, height, and other characteristics such as ASA status, diagnosis, type of craniotomy, incision type, duration of surgery, and duration of anesthesia were statistically insignificant (p> 0.05) (Table-I).

**Fig.1:**
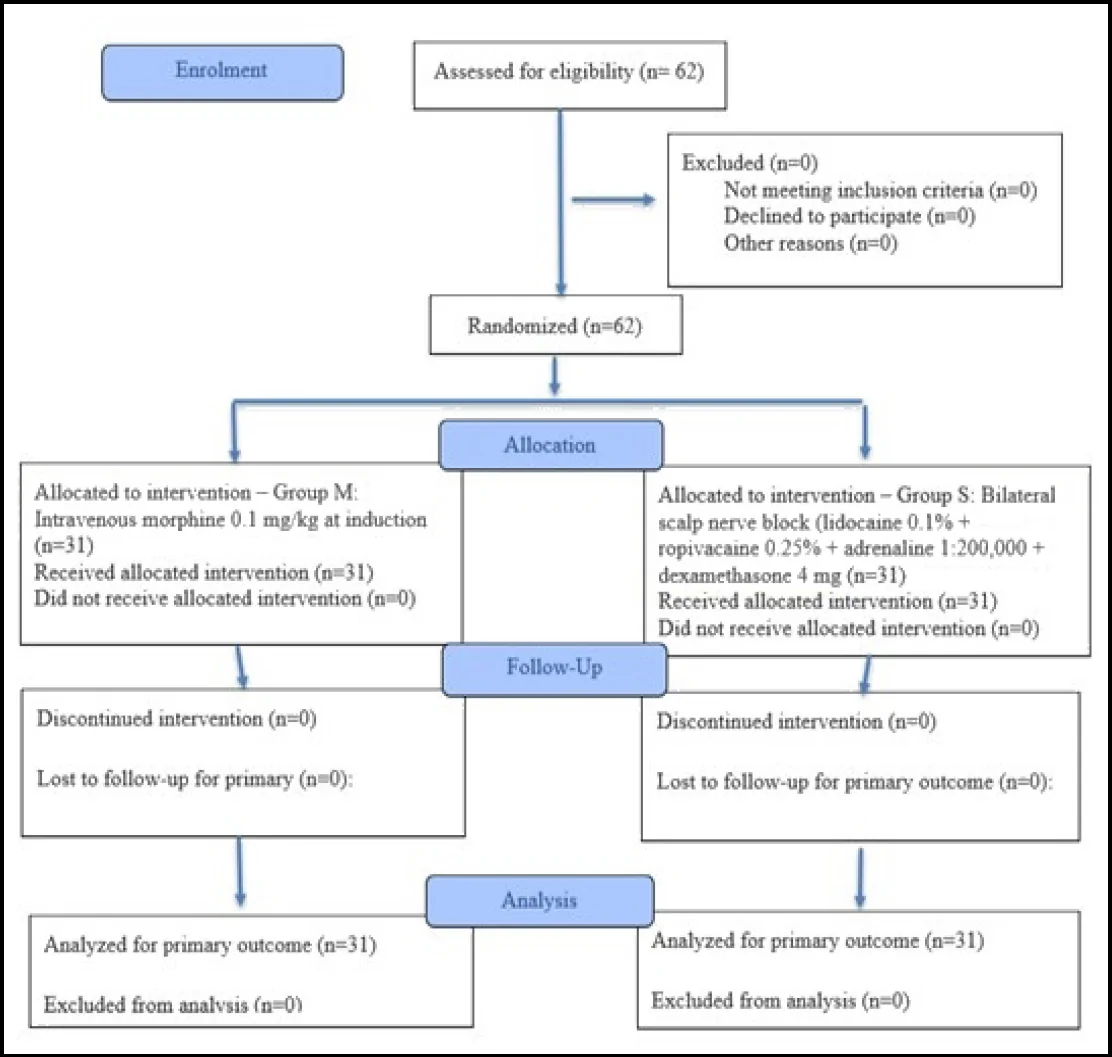
CONSORT Flow Diagram.

**Table-I:** Demographic Data of the Study Participants

Variable	Group S (SNB) n=31	Group M (Morphine) n=31	P value
Age (years) *	44.3 ± 11.9	44.6 ± 14.5	0.932
Sex M/F ^#^	10 (32.3) / 21(67.7)	15 (48.4) /16 (51.6)	0.300
ASA I/II/III ^#^	2 / 23 / 6	3 / 21 / 7	0.845
Weight (kg) *	72.5 ± 14.2	69.2 ± 12.5	0.338
Height (cm) *	164 ± 9.8	169 ± 10.6	0.085
Duration of surgery (min) *	255 ± 81.6	247 ± 81.3	0.698
Duration of anesthesia (min) *	339 ± 93.6	325 ± 69.5	0.504
***Diagnosis***			
Brain tumor ^#^	23 (74.2)	23 (74.2)	
Meningioma ^#^	7 (22.6)	6 (19.4)	1.000
Intracranial aneurysm ^#^	1 (3.2)	1 (3.2)	
Hemangioma ^#^	0	1 (3.2)	
***Type of Craniotomy***			
Bifrontal ^#^	1 (3.2%)	0 (0%)	
Frontal ^#^	11 (35.5%)	7 (22.6%)	
Frontoparietal ^#^	6 (19.4%)	1 (3.2%)	
Frontotemporal ^#^	5 (16.1%)	7 (22.6%)	0.112
Parietal ^#^	5 (16.1%)	10 (32.2%)	
Temporal ^#^	2 (6.5%)	6 (19.4%)	
Temporoparietal ^#^	1 (3.2%)	0 (0%)	
***Type of Incision***			
Curvilinear ^#^	1 (3.2%)	0 (0%)	
Hockey Stick ^#^	1 (3.2%)	1 (3.2%)	
Linear ^#^	5 (16.1%)	4 (12.9%)	
Midline ^#^	9 (29%)	5 (16.1%)	0.383
Paramedian ^#^	2 (6.5%)	7 (22.6%)	
Pterional ^#^	13 (41.9%)	13 (41.9%)	
Frontal Extended ^#^	0 (0%)	1 (3.2%)	

***Note:*** Data presented as mean ± SD. p-Values significant at < 0.05.

The baseline vitals, heart rate, mean blood pressure (MBP), systolic blood pressure (SBP) and diastolic blood pressure (DBP), in both groups were equivocal (Fig.2). Following the induction of anesthesia, heart rate was almost similar in both study groups at most of the interval timings, except a slightly elevation was recorded in Group-S during the period of intense surgical stimulation (head pinning, incision, bone dissection and dural manipulation), although statistically it was not significant (Fig.2).

**Fig.2:**
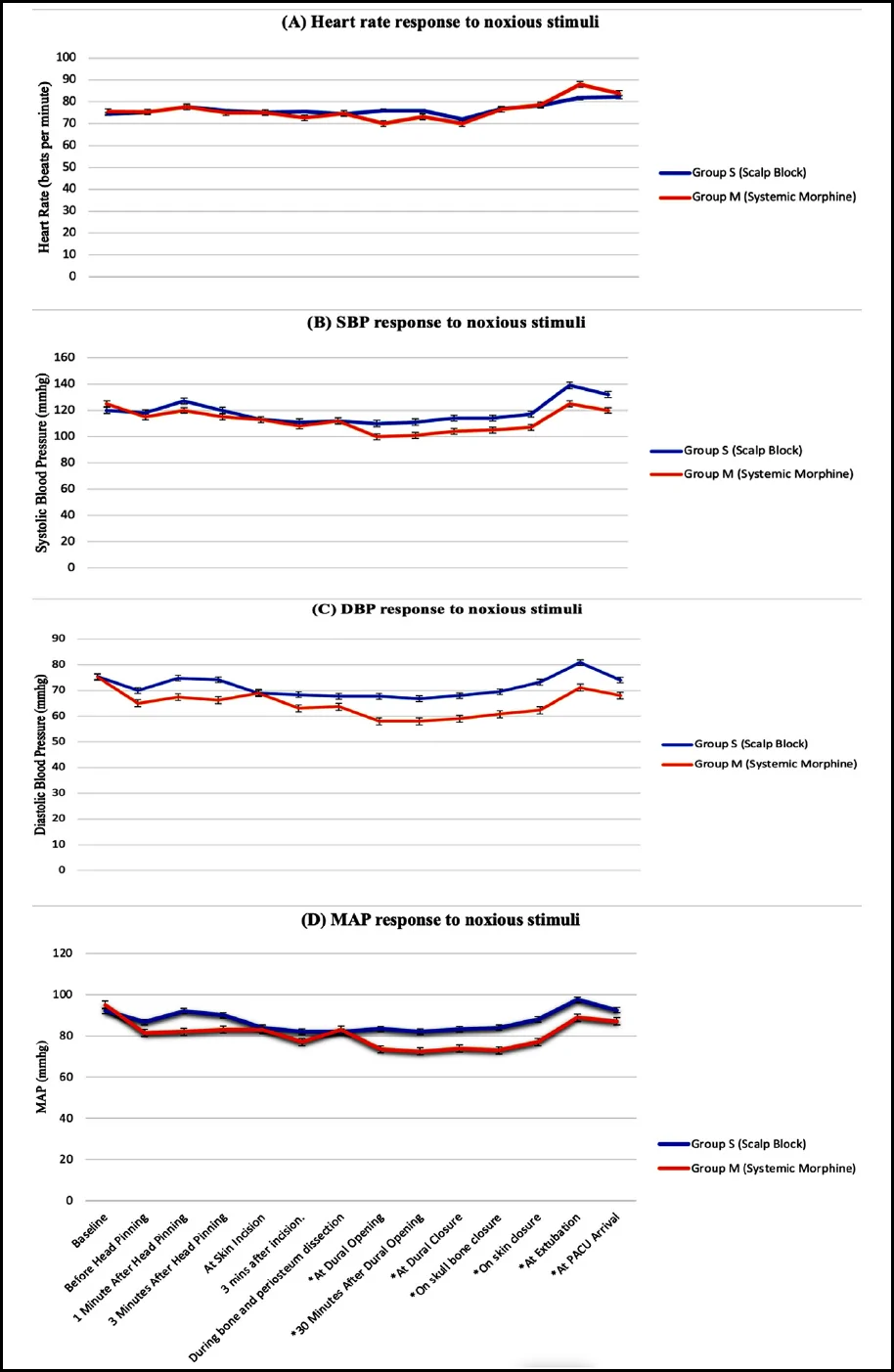
(A) Mean Heart Rate (HR/PBM), (B) Mean Systolic Blood Pressure (SBP, mmHg), (C) Mean Diastolic Blood Pressure (DBP, mmHg), (D) Mean Arterial Pressure (MAP, mmHg), response to various noxoius stimuli ***Note:*** *Level of significance between Group SNB and Group M, P<0.05.

The SBP was significantly lower in Group-M, at dural opening, at 30 minutes, dural closure, skull bone closure, skin closure, at extubation and on PACU arrival (Fig.2). Moreover, the DBP was static at skin incision and during bone dissection in both groups, but it has shown significant lower readings in Group-M, at one minute after pinning, three minutes after pinning, dural opening, at 30-minutes, dural closure, skull closure, skin closure, extubation and on PACU arrival (Fig.2). The MAP was statistically higher in Group-S, compared to Group-M at dural opening, 30-minute interval, dural closure, skull closure, skin closure, extubation and PACU arrival (Fig.2).

The study was intended to apply morphine PCIA to all patients but due to opioid supply limitation the 15/31 patients (48.4%), received in group SNB and 21/31 (67.7%), in Group-M. The PCIA morphine boluses were categorized into 1-3 boluses and recorded at given intervals that found to be greater delivery in Group-M compared to group SNB at almost all time intervals (Table-II). The intraoperative rescue analgesia (fentanyl), requirement was also relatively higher in Group-M compared to Group-S, though it was statistically insignificant (Table-III). The VAS pain scores were comparable during the immediate PACU stay. However, from two hours onward up to 24 hours, Group-S demonstrated significantly lower VAS scores compares to Group-M. (Fig.3). The sedation (Table-IV), and nausea/vomiting scores were almost similar in both groups (Table-V), and no post-operative complications were observed in the recruited patients until the time of hospital discharge.

**Table-II:** Number of PCIA Boluses In Both Study Groups

	SNB (N=31)	M (N=31)	P-value
***On PACU arrival***			
0	15 (48.4%)	20 (64.5%)	0.198
NO PCIA	16 (51.6%)	10 (32.3%)	
3	0 (0%)	1 (3.2%)	
***At 30 mins interval***			
0	9 (29.0%)	16 (51.6%)	0.285
1	4 (12.9%)	4 (12.9%)	
2	2 (6.5%)	1 (3.2%)	
NO PCIA	16 (51.6%)	10 (32.3%)	
***At 1 hour interval***			
0	7 (22.6%)	10 (32.3%)	0.498
1	6 (19.4%)	8 (25.8%)	
2	2 (6.5%)	3 (9.7%)	
NO PCIA	16 (51.6%)	10 (32.3%)	
***At 2 hours interval***			
0	9 (29.0%)	8 (25.8%)	0.353
1	4 (12.9%)	8 (25.8%)	
2	2 (6.5%)	4 (12.9%)	
NO PCIA	16 (51.6%)	10 (32.3%)	
3	0 (0%)	1 (3.2%)	
***At 4 hour interval***			
0	12 (38.7%)	12 (38.7%)	0.206
1	3 (9.7%)	5 (16.1%)	
NO PCIA	16 (51.6%)	10 (32.3%)	
2	0 (0%)	3 (9.7%)	
3	0 (0%)	1 (3.2%)	
***At 6 hour interval***			
0	15 (48.4%)	13 (41.9%)	0.0176
NO PCIA	16 (51.6%)	10 (32.3%)	
1	0 (0%)	6 (19.4%)	
2	0 (0%)	1 (3.2%)	
3	0 (0%)	1 (3.2%)	
***At 12 hour interval***			
0	14 (45.2%)	16 (51.6%)	0.145
1	1 (3.2%)	5 (16.1%)	
NO PCIA	16 (51.6%)	10 (32.3%)	
***At 18 hour interval***			
0	14 (45.2%)	17 (54.8%)	0.181
1	1 (3.2%)	4 (12.9%)	
NO PCIA	16 (51.6%)	10 (32.3%)	
***At 24 hour interval***			
0	14 (45.2%)	19 (61.3%)	0.384
1	1 (3.2%)	2 (6.5%)	
NO PCIA	16 (51.6%)	10 (32.3%)	

***Note:*** Data presented as total number of patients (n), p-Values significant at < 0.05

**Table-III:** Fentanyl requirement (rescue analgesia).

Group	Mean ± SD	Minimum	Maximum	P-value
S (SNB)	0.15 ± 0.23	0.0	0.5	0.486
M (Morphine)	0.19 ± 0.31	0.0	1.0	

***Note:*** Data presented as mean ± SD. p-Values significant at P < 0.05.

**Fig.3:**
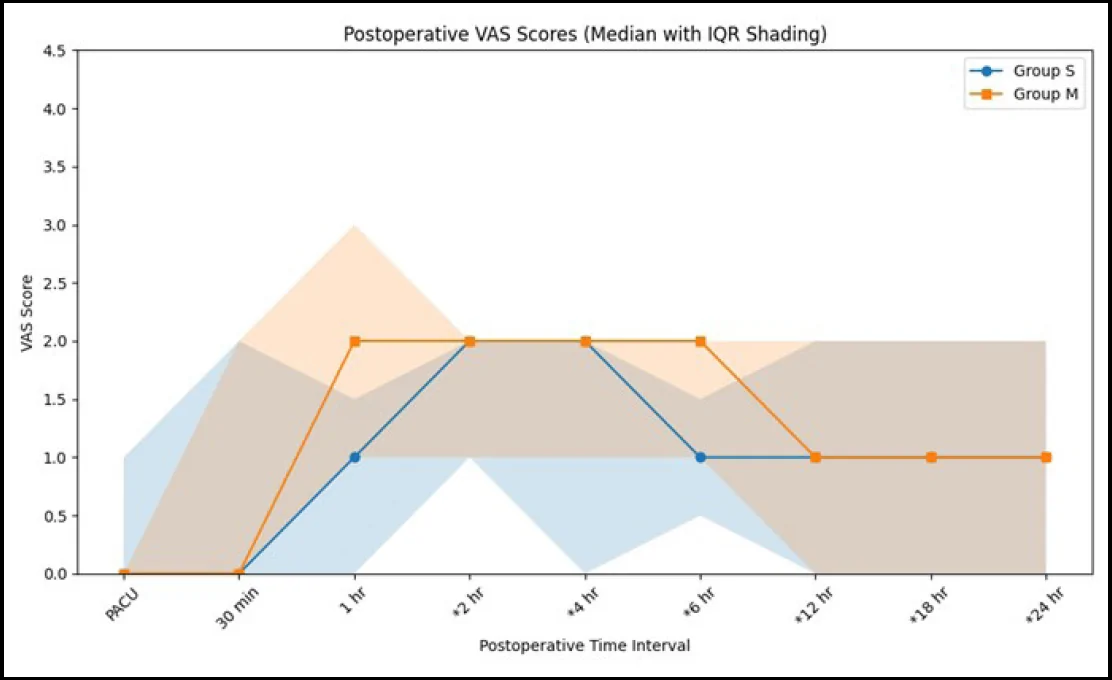
Visual Analogue Scale (VAS) Pain Score.

**Table-IV:** Sedation scores.

Group	Group S (SNB)				Group M				P-value
Score / Time	1	2	3	4	1	2	3	4	
PACU	6	23	2	0	5	23	3	0	1.000
0.5 hour	11	18	2	0	8	21	2	0	0.836
1 hour	19	10	2	0	16	14	1	0	0.565
2 hours	25	5	1	0	24	6	1	0	1.000
4 hours	27	3	1	0	25	6	0	0	0.473
6 hours	27	4	0	0	28	3	0	0	1.000
12 hours	27	4	0	0	29	2	0	0	0.671
18 hours	28	3	0	0	29	2	0	0	1.000
24 hours	29	2	0	0	29	2	0	0	1.000

***Note:*** *Level of significance between Group SNB and M, P<0.05.

**Table-V:** NAUSEA/Vomiting Scores.

Group	Group S (SNB)					Group M			
Score / Time	0	1	2	3	0	1	2	3	P- value
PACU	25	4	2	0	25	6	0	0	0.512
0.5 hour	28	3	0	0	28	3	0	0	1.000
1 hour	27	3	1	0	30	1	0	0	0.354
2 hours	31	0	0	0	29	1	1	0	0.492
4 hours	31	0	0	0	29	2	0	0	0.492
6 hours	31	0	0	0	29	2	0	0	0.492
12 hours	31	0	0	0	31	0	0	0	1.000
18 hours	31	0	0	0	31	0	0	0	1.000
24 hours	31	0	0	0	31	0	0	0	1.000

***Note:*** Data presented as total number of patients (n), p-Values significant at < 0.05.

## DISCUSSION

The positive finding of our study is the hemodynamic stability recorded with the scalp nerve block to the various noxious stimuli in supratentorial craniotomy surgeries that underwent almost every type of cranial incision and craniotomy approach. However, morphine has also shown promising intraoperative effects but with marginal hemodynamic variability compared to the SNB group. Furthermore, a noticeable finding includes the pain score on PACU arrival and up to 24 hours, which was well managed with scalp nerve block patients, though the PCIA morphine availability caused limitation, but it was relatively more available for the Group-M than for the patients who fell in the group SNB. Moreover, the rescue fentanyl requirement was relatively higher in Group-M than in the Group SNB. Along with that, our study failed to signify that the sedation, nausea, and vomiting incidence was different but instead were equivocal in both study groups.

Our study used the mixture of 0.1% lidocaine and 0.25% ropivacaine with 1:200,000 adrenaline and 4-mg dexamethasone, which provided both pharmacokinetic and clinical advantages in scalp nerve blockade. Due to lidocaine’s quick onset of action, nociceptive input is immediately attenuated during times of intense stimulation. Author preferred ropivacaine, a long-acting amide local anesthetic, which has a better cardiotoxicity profile; it extends the duration of analgesia and helps maintain postoperative pain management. Along with that, dexamethasone and adrenaline were used as adjuvants to prolong the duration of action of SNB.

Effective management of the intense sympathetic response caused by perioperative noxious stimuli during craniotomy surgeries continue to be a significant anesthetic challenge. These nociceptive stimuli have the potential to disrupt cerebral perfusion and intracranial dynamics.^4^ General and regional anesthesia work in harmony to provide a number of advantages. By acting on the pain pathway at several levels, multimodal techniques augment the analgesic efficacy while reducing the need for high-dose opioids.

Literature also supports our study findings for the scalp nerve block. Geze et al^5^. demonstrated the scalp block significantly attenuates the hemodynamic and stress response to skull-pin placement during craniotomy. Another study by Jayaram et al. reported the regional scalp anesthesia presented an effective adjunct to systemic analgesia, provided targeted blockade of nociceptive input from the richly innervated scalp.^9^ and, Vadivelu et al.^10^ also highlighted the importance of multimodal analgesia in neurosurgery, emphasizing that combination of regional techniques with systemic analgesics reduces opioid-related adverse effects and enhances the recovery.

Similarly, Nguyen et al. reported that scalp nerve block decreases postoperative pain severity following craniotomy.^13^ Ayoub et al. further showed that scalp block provided transitional analgesia similar to when compared with systemic morphine.^11^ These findings were reinforced by a systematic review and meta-analysis by Guilfoyle et al., which confirmed that regional scalp block improves early post-craniotomy analgesia without significant safety concerns.^14^ Bombardieri et al. also stated that SNB is low-risk, technically simple, and effective across many neurosurgical procedures.^15^ Zhang et al. similarly found that SNB also significantly improves the quality of short-term postoperative rehabilitation in patients.^16^ More recent randomized trials support these findings. Moharari et al. showed that scalp block improved intraoperative analgesia and reduced opioid consumption during elective craniotomy under nociception-index monitoring,^17^ and Xiong et al. reported comparable benefit of pre-incision scalp block in paediatric craniotomy.^18^ A recent meta-analysis of enhanced recovery after surgery (ERAS) protocols in craniotomy, regional scalp blockade was also found to be a component of treatment linked to shorter hospital stays and less postoperative nausea and vomiting.^19^ Local data even though extremely limited also demonstrates that pre-incision, scalp nerve blocks have shown significant benefits in terms of managing postoperative pain.^20^

It should also be acknowledged that the evidence for scalp nerve block is not uniformly favorable. Current PROSPECT recommendations for craniotomy treat regional techniques such as scalp block or incision-site infiltration as one of several interchangeable components of a multimodal regimen that also includes paracetamol, NSAIDs, and dexmedetomidine, rather than as a necessary intervention.^6^ The incremental benefit of scalp nerve block may therefore be most apparent where systemic opioids are the principal alternative, such as in our setting, rather than where a full non-opioid multimodal regimen is already routinely available.

### Limitations

First, it is a single-center study which may limit the generalizability. Secondly, limited availability of PCA morphine for postoperative pain management and quantification of total opioid consumption in the first 24 hours caused challenges in study generalizability, although it supports the previously published data of recurrent narcotic shortages in our lower-middle-income country settings.^21^ Thirdly, our study failed to standardize the postoperative pain management during the shortage of PCIA morphine. Fourth, our study recorded non-invasive hemodynamic variables until the invasive line was placed.

## CONCLUSION

Pre-incisional scalp nerve block showed promising analgesic effects by stabilization of intraoperative hemodynamics in response to various noxious stimuli in supratentorial craniotomy surgeries. Along with that, it has shown superiority for postoperative analgesia, without risking sedation, nausea, and vomiting in the first 24 hours. Moreover, the SNB can be considered a substitute for intravenous morphine, especially in times of unavailability of narcotics in middle-income countries (LMICs).
